# Robotic misinformation in dementia care: emotions as sense-making resources in residents’ encounters with robot animals

**DOI:** 10.3389/fsoc.2024.1354978

**Published:** 2024-04-08

**Authors:** Marcus Persson, Elin Thunman, Clara Iversen, David Redmalm

**Affiliations:** ^1^Institution of Behavioral Sciences and Learning, Department of Education and Sociology, Linköping University, Linköping, Sweden; ^2^Department of Sociology, Uppsala University, Uppsala, Sweden; ^3^Department of Social Work, Uppsala University, Uppsala, Sweden; ^4^Division of Sociology, School of Health, Care and Social Welfare, Mälardalen University, Västerås, Sweden

**Keywords:** misinformative robots, animacy judgment, dementia care, residents, care workers, sense-making, emotions, interactionist sociomaterialism

## Abstract

Robot animals, designed to mimic living beings, pose ethical challenges in the context of caring for vulnerable patients, specifically concerning deception. This paper explores how emotions become a resource for dealing with the misinformative nature of robot animals in dementia care homes. Based on observations of encounters between residents, care workers, and robot animals, the study shows how persons with dementia approach the ambiguous robots as either living beings, material artifacts, or something in-between. Grounded in interactionist theory, the research demonstrates that emotions serve as tools in the sense-making process, occurring through interactions with the material object and in collaboration with care workers. The appreciation of social robots does not solely hinge on them being perceived as real or fake animals; persons with dementia may find amusement in “fake” animals and express fear of “real” ones. This observation leads us to argue that there is a gap between guidelines addressing misinformation and robots and the specific context in which the technology is in use. In situations where small talk and play are essential activities, care workers often prioritize responsiveness to residents rather than making sure that the robot’s nature is transparent. In these situations, residents’ emotional expressions serve not only as crucial resources for their own sense-making but also as valuable indicators for care workers to comprehend how to navigate care situations.

## 1 Introduction

Studies on technology and information disorder have frequently centered on media and news production. In this context, emotions are recognized to play a pivotal role, such as through expressions of aggression, fear, and outrage when individuals share fake content with each other (Taddicken and Wolff, 2020; Serrano-Puche, 2021). If the intent of creating false information is to cause harm, it is usually referred to as disinformation (Wardle and Derakhshan, 2018, p. 20). A remedy for addressing disinformation is information validation and training to see through lies (de Oliveira and Leitão, 2022). Although this study takes on a radically different area – robot animals in dementia care – the discussed problems and solutions are remarkably similar. Robot animals are designed to mimic living animals, meaning that information about their nature is false or hidden, albeit not with intention to cause harm. Consequently, the robot animals have a built-in potential for misinformation (Wardle and Derakhshan, 2018, p. 20), which is the focus of this paper. The ambiguous nature of the robot, whether perceived as a machine or a living animal, is often regarded as a problematic feature, requiring a response grounded in transparency and information (Blackman, 2013; Vandemeulebroucke et al., 2018).

The aim of the paper is to explore dementia care residents’ emotional orientations to robotic misinformation in interaction with care workers. Instead of treating emotions as a threat evoked by false information, we will discuss emotions as part of rational conduct (Putnam and Mumby, 1993; Bergman Blix and Wettergren, 2018), even key resources for accomplishing professional care in uncertain conditions (*cf.* Minissale, 2023). Drawing on the insights of the practical use of robot animals in dementia care, we discuss how emotions become a resource for dealing with ambiguity related to misinformation. In doing so, the paper represents an initial attempt to explore new ways of thinking about emotion and misinformation.

As part of a larger trend of implementing digital tools in elderly care (Frennert and Östlund, 2018), robot animals are used for facilitating care for older persons, for instance by helping care workers to calm agitated patients or to promote activities, such as small talk and cuddling. In social robotics, the principle of transparency has been promoted, stating that robots’ design should reflect their actual capacities (Złotowski et al., 2020). This may be particularly relevant in dementia care, where patients may face difficulties navigating reality (Örulv and Hydén, 2006; Perach et al., 2020); care without deceit is considered a vital part of treating patients with dignity (Blackman, 2013; Sharkey and Sharkey, 2020). However, robot animals are built to give the impression that the user is interacting with a living being: they have life-like fur, sound, and movements and are usually equipped with sonic or tactile sensors that enable them to respond to users’ talk or touch (Barber et al., 2021). The way these robots invite the user into playful interaction may make them seem like a perfect tool for care workers, especially as previous research has acknowledged the value of play and pretense in dementia care (e.g., Kontos, 2004). Yet, the robots’ lifelike design might come in conflict with transparency and information (Sætra, 2021). Thus, the question of how to deal with robotic misinformation comes to the fore in dementia care.

Ambiguity tends to be analyzed as a consequence of lack of clarity or irreconcilable contradictions, influencing the emotional labor needed in different situations (Styhre et al., 2002). It has been described as leading to confusion, ambivalence, and cynicism (Meyerson, 1990) but also as a potential source of curiosity and joy (Frezza et al., 2022). Translated to an interactionist framework, the misinformative nature of the robot can be understood as introducing a disruption in perceived reality and, as such, the need for rekindling sense-making (Mead, 1972, p. 3–25). Mead (1972, p. 79) characterizes such problematic situations in terms of “resistance” experienced by the individual. Resistance indicates a disruption in customary actions that compels the actor to pause, become aware of the problem, and endeavor to overcome it (Rosenthal and Bourgeois, 1991). Our analytical point of departure is that emotional work related to robotic misinformation is both done by residents and care workers. Residents manipulate and react to robots in interaction with one another and with care workers, who are responsible for offering support and helping the users to make sense of the existential ambiguity of the robot. According to this view, users’ emotional responses to the robotic ambiguity are thus interactional phenomena situated in the triadic relation between residents, care workers, and robots.

The structure of the paper unfolds as follows: Initially, we review existing research related to the utilization of robot animals in dementia care. Then we introduce our theoretical framework, which is rooted in interactionist sociomaterialism which emphasizes the social dynamics between humans and material objects. In the methodology section, we describe our ethnographic approach to studying robots within dementia care homes. The ensuing sections delve into the presentation of our analyses of the empirical findings. These illustrate the profound involvement of various emotions in the sense-making efforts of demented residents when interacting with the ambiguous robot animals. Finally, within the discussion section, we summarize our results and contemplate their implications for the practical implementation of robotic animals in dementia care.

## 2 Studying robots in social context

In studies of social robots in care for older people, there is a predominant biomedical focus on the impact of robots on patients’ health and well-being. Much of the research on robot animals aims to identify the benefits of these robotic companions by measuring, for example, changes in the quality of life and behavior of users, particularly people living with dementia (Abbott et al., 2019). Emotions, in this context, are often discussed as expressions of symptoms of the illness, such as increased agitation, fear, anxiety, anhedonia, and aggression (see Cerejeira et al., 2012; Ismail et al., 2018). Studies have indicated that the use of social robots can have a soothing and calming effect on the residents in response to such illness related emotions (Chang and Šabanović, 2015; Birks et al., 2016; Robinson et al., 2016; Moyle et al., 2018).

In contrast to the biomedical perspective, scholars with a psychosocial orientation argue that “negative” emotions can be interpreted as affective signs rather than symptoms, revealing the unmet needs of the residents. Frezza et al. (2022) highlight that external circumstances often trigger negative emotions in people with dementia, such as anger, fear, and sadness. Some of these circumstances can be related to the absence of certain elements in their lives, such as social relationships and physical contact (Verdelho and Gonçalves-Pereira, 2017). Others can be linked to the inability to perform actions, whether cognitive or physical, leading to feelings of clumsiness, loss of control, and decline. Issues with technology can also be a source of frustration (Preston et al., 2007). For example, when confronted with a robot animal, negative emotions may emerge related to the need to understand the nature (“What is it?”) and capabilities (“What can it do?”) of the robot. The inability to make sense of the robot may stir up emotions of frustration, anxiety, or even fear (Moyle et al., 2018).

Several studies have observed how residents often treat robot animals as they would real pets, displaying affection through actions like hugging, petting, kissing, and stroking (Robinson et al., 2016). Verbal responses from residents also indicate that they see these robots as living creatures (Giusti and Marti, 2008; Chang and Šabanović, 2015). However, some studies indicate that residents can develop an “emotional attachment” to the robot animals, fully aware that they are not “real” (Gustafsson et al., 2015; Robinson et al., 2016). Thus, the subjective interpretation of robots and emotional responses is complex and requires further investigation.

In this paper we adopt a relational approach which involves understanding the residents’ use of robot animals in social situations that encompass material objects and other social subjects (Robinson et al., 2016; DeFalco, 2020). Previous studies have shown that social robots demand involvement from care workers to engage users (Chevallier, 2022; Persson et al., 2023). For instance, the care staff may need to participate in conversations (Chang and Šabanović, 2015) to ensure that the robot is accessible at the right moment (Jung et al., 2017; Moyle et al., 2018), and to “stage” it by carrying it in a particular manner, discussing it, and sometimes physically guiding users on how to handle it (Pfadenhauer and Dukat, 2015). In this regard, studies have shown how robot animals can serve as an “icebreaker” between staff and residents, with staff members often “joking and laughing” with residents about the robot (Robinson et al., 2016). However, Chevallier (2022) also shows that forceful attempts to engage residents, including physical cues, can backfire and result in annoyance and anger (see also Persson et al., 2024).

While much of this research on interactions and emotions departs from the perspective of care workers, it clearly demonstrates the presence and importance of residents’ emotions in the triadic interaction between the resident, robot, and care worker. Therefore, the current study contributes insights into how the ambiguity of biomimetic robot animals is managed in dementia care.

## 3 Interactionist sociomaterialism

Our theoretical framework is grounded in interactionist sociomaterialism dating back to George Herbert Mead. The theory posits that reality is pragmatic; it is located not in the cognitive realm of the human mind, nor in the external world “out there.” Rather, reality is located in the “act” that involves both the ongoing perceptual relations that emerge between human consciousness and the surrounding social and material environment (Puddephatt, 2005, p. 364). Mutual understanding arises as individuals take on each other’s roles and attitudes toward a phenomenon or an object (Hewitt, 1976). For instance, a chair becomes functional when an individual interacts with it by sitting on it. Its social or shared meaning only materializes when two or more individuals use it in a similar way and refer to it as a “chair,” treating it as a “significant symbol” (Mead, 1967, p. 46, 71f) in their social communication. Communication through significant symbols, whether verbal or non-verbal gestures, creates a shared world of symbolic meanings (Ashmore et al., 1994; Preda, 1999).

Once a shared understanding of reality is established, individuals develop trust that the world will respond to their actions as expected. However, reality does not always conform to these anticipations. When the customary flow of actions is disrupted due to a “problematic situation,” the need for sense-making is rekindled (Mead, 1972, p. 3–25). Mead (1972, p. 79) characterizes such problematic situations in terms of “resistance” experienced by the individual. Resistance indicates a disruption in customary actions that compels the actor to pause, become aware of the problem, and endeavor to overcome it (Rosenthal and Bourgeois, 1991). The reality, as perceived by the individual, is questioned when problems surface. To resolve these issues and reaffirm the world’s “realness,” individuals must reconstruct their established hypotheses about the world. This involves testing new hypotheses through interaction with the object and other individuals. If the new hypothesis proves successful, the individual can continue until new problems arise (Mead, 1972, p. 280).

Applied to our subject of study, the introduction of a social robot as an unfamiliar material object may lead to experiences of inhibition, prompting individuals with dementia to “resolve the problem” by defining what the robot is and what function it serves for them. Hence, the meaning of the robot must be constructed in interaction with the object and in collaboration with others to establish a shared understanding of the robot’s existence and function.

Experiencing resistance compels the individual to pay attention to the problem and make efforts to overcome the resistance (McCarthy, 1984; Puddephatt, 2005). Consequently, experiences of resistance trigger reflection (Mead, 1972, p. 79). Interactionist research (Joas, 1985; Engdahl, 2005) has after Mead emphasized that experiences of resistance not only give rise to heightened awareness in the form of reflection but also evoke emotions (Stein et al., 2015). For example, in a study on road rage, Katz (1999) elucidates the intense emotions experienced by drivers during traffic jams as a form of resistance. These emotions can be expressed through verbal and non-verbal gestures. Katz (1999, p. 317ff) suggests that emotions manifest in situations where the intertwining between the self and the world becomes problematic. A crisis occurs when an individual lacks a habitual shared understanding of what a particular phenomenon or thing is and how it should be defined. Katz argues that emotions are crucial resources for restoring the world (and the self) as meaningful in problematic situations. He describes “emotional moments as sense-making in everyday social interaction” (p 324). Therefore, emotional orientations should be considered vital manifestations of agency and the individual’s capacity for creative, problem-solving actions to address inhibitory experiences encountered in daily life (Joas, 1996).

Incorporating the concept of resistance and the role of emotions as resources in sense-making, we will explore the interactions between individuals with dementia and robot animals.

## 4 Ethnography in dementia care

To study residents’ emotions in encounters with robot animals, this paper draws on qualitative data collected at five dementia care homes in Sweden. The participating facilities were selected due to their experience of using robot animals in the care work. The robots in the study are of the same brand and relatively inexpensive, designed to mimic real cats and dog. The cat is equipped with pressure sensors under its fur, allowing it to react to touch, triggering data protocols that result in both movement and sound. For instance, it can rotate its upper body backward, raise one paw to its face, meow, and purr. The dogs also respond to touch and feature sound sensors that enable them to turn their head and bark when users interact with them. Additionally, the dogs are equipped with machinery that simulates a “heartbeat”.

The ethnographic fieldwork (*cf.* Hammersley and Atkinson, 2007) was conducted with residents and care workers in five dementia care settings in Sweden. Observations and interviews were conducted by one researcher at a time, and the duration spanned from a one-day visit to smaller care homes, to week-long visits to larger care homes. In total, the research team spent about 100 h at care homes. The role of the visiting researcher can be understood as “participating observer” (Emerson et al., 2011), which means that the researcher followed the care workers when they carried out their daily round of activities.

The study received approval from the Ethical Board in Sweden (Dnr 2020-04661). Informed consent was obtained from both care workers and residents before commencing fieldwork. As argued by Hellström et al. (2007), ethical considerations pose challenges in research involving individuals living with dementia. The principle of information necessitates that researchers inform all participants about the study, their role in the project, and the voluntary nature of their participation, emphasizing their right to withdraw at any point without specifying reasons. Moreover, following the recommendations of McKeown et al. (2010), we ensured that participants living with dementia were regularly informed about the study and their options to participate or abstain during each interaction.

Documentation was carried out through audio-recorded conversations with care workers and residents which were then transcribed verbatim, and field notes consisting of rich descriptions of the environment, the people, and the events that we encountered (Hammersley and Atkinson, 2007). Audio-recorded conversations were conducted with 44 care workers and 15 residents during the visits at the care homes (Merton et al., 1956). We also conducted video-recorded observations of interactions involving robot animals, residents, and care workers at two dementia facilities. In total, we recorded about 200 min of interaction, which was transcribed in detail including embodied and verbal practices.

The analysis was based on thematic analysis, which means that we read through transcripts looking for recurrent and meaningful themes (*cf.* Braun and Clarke, 2006). An essential aspect of the analysis involved reading the transcripts while considering the field notes and video-data. Collecting various data sources enabled us to achieve a comprehensive understanding of how the participants emotionally responded to social robots. The interview transcripts, field notes, and video-data were meticulously coded, focusing on the residents’ emotional expressions during interactions with the robots and care workers. This coding process involved analyzing the residents’ positioning in various situations, particularly when the misleading nature of the robots became apparent. The codes of emotions were subsequently compared and grouped into categories with similar meaning content (Hayes, 1997). These analytical categories were thoroughly discussed and refined to ensure they were mutually exclusive and devoid of overlapping meanings (*cf.* Aspers, 2007). Each category’s definition was further developed and clarified in alignment with our theoretical framework (*cf.* Braun and Clarke, 2006). Finally, we validated the categories by applying them to empirical excerpts and comparing them with our comprehensive interpretations of the dataset.

## 5 Residents’ encounters with robot animals

Below we will account for the findings in three sections. First, we focus on how residents approach the resistance robot animals offer in terms of their existential ambiguity and how they, together with care workers, perform problem solving to overcome the experienced resistance. Thereafter, we take a closer look at the residents’ different emotional orientations in relation to the misinformative design of the robot. We first discuss positive emotional orientations and then negative orientations in relation to whether the residents make the animacy judgment about the robot as a living animal or as a non-living artifact (Castro-González et al., 2016; Kim et al., 2019; Barber et al., 2021).

### 5.1 Resistance and problem-solving

When people encounter social robots, they need to establish the robots’ ability to understand and respond to human action (Tuncer et al., 2023). When a person with dementia meets a robot animal, this work may be especially important, given the potential uncertain relation to reality as well as the built-in ambiguous element in the robot animal. This section focuses on residents’ initial actions when encountering a robot animal trying to figure out what it is and how to approach it.

This initial action of sensemaking is illustrated in the following interaction moment (Home 3) in which Flora (care worker) is introducing Elisabeth (resident) to a robot cat without defining it. Flora says: “Have you seen, Elisabeth!,” and shows her the robot cat. Elisabeth looks at it with astonishment, and asks: “Is it alive?” In Meadian terms, Flora’s introduction of the robot animal can be understood to give rise to a disruption in customary actions that compels the actor to pause, become aware of the problem, and endeavor to overcome it. In such terms, Elisabeth can be understood as encountering resistance in her natural approach to the world, evident in her astonished look and her question if the robot cat is real or not. Without any verbal cues from the care worker as to what the nature of this thing is, Elisabeth starts exploring the robot visually and physically, touching and petting it. With Mead, she tries to “solve the problem” by defining what the robot is, what function it should be given, by interacting with it. This exploration is made possible as Flora has made the robot available for gaze and touch.

Flora puts the robot down on the table in front of Elisabeth and they explore—verbally and physically—the robot together. Attempting to make sense of the robot cat and figure out its capabilities, Elisabeth does not just use the interaction with the robot animal. She also uses the interaction with Flora as a resource to define what the robot is, and by that overcoming the resistance it offers. This is apparent in her asking: “It does not pee inside, does it?.” The care worker responds to this question as concerned with worry by reassuring her that “No, he does not pee inside” And by doing so, Flora simultaneously ascribed gender (“he”) and agency to the robot (“does not pee”), but still neither defining the object as a real nor as a robot animal in the interaction with the resident. Thus, by reassuring rather than offering detailed information about the robot, Flora deals with what is conveyed emotionally in Elisabeth’s question.

The conversation continues and after a while Elisabeth turns her attention directly to the robot, and the care worker assumes a more supporting role. For example, Elisabeth, now directly oriented toward communicating with the robot itself, asks it: “Are you hungry? Do you want food?.” By asking these questions, Elisabeth indicates that she is about to solve the problem of what the robot is by treating it as a real animal. The care worker replies, “No he does not eat any food,” which treats the robot as real using a person reference (“he”) at the same time as she reveals information about the robot indicating that it does not have the needs of a living being. This answer thus offers enough information for Elisabeth to be able to proceed with the interaction but does not deal with the reality of the robot.

In her further interaction with the inhibiting object, Elisabeth pats the robot and touches it where the pressure sensors are placed, which triggers the robots’ programed responses. It meows and stretches. Elisabeth gives off a sound of astonishment in response to the robots’ movement: “Oh my! How he stretches!” thus adopting Flora’s way of referencing the robot. Here, the robot’s activity is responded to as something engendering surprise and wonder rather than analytic examination. The robot’s movement spurs her on to further interaction and patting. Now Elisabeth and the robot interact without support from Flora. Each time the robot meows or makes a bodily movement, Elisabeth responds, for instance by imitating the robot cat’s purring, or by engaging in small talk such as: “yes, do you like it when I do this?,” while stroking the robot behind the ears.

Drawing on Mead, we can understand this interaction as if Elisabeth has overcome the reality hiccup—the built in resistance in the biomimetic robot’s ambiguousness—offered by the robot’s introduction in her world. While we have not seen a clear verbal definition of the robot with an accompanying embodied stance, the pragmatic orientation of the robot shows that it is sufficiently real to be included in progressing interaction. The lack of informational clarity about the robot’s existential status and abilities is downplayed in favor of an emotional orientation toward the robot.

If a resident, by contrast, does not manage to overcome the robot’s momentary challenge against reality via interaction supported by care workers, it will likely be put away. As another care worker (Marie, Home 2) says: “We have someone we thought you could use it (the robot) with, but it does not really work on her. She does not want it… or does not really understand what it is.” Put in theoretical terms, the resident has not solved the “reality crisis” of the robot animal; she has not overcome the resistance it offered.

**Figure 1:**
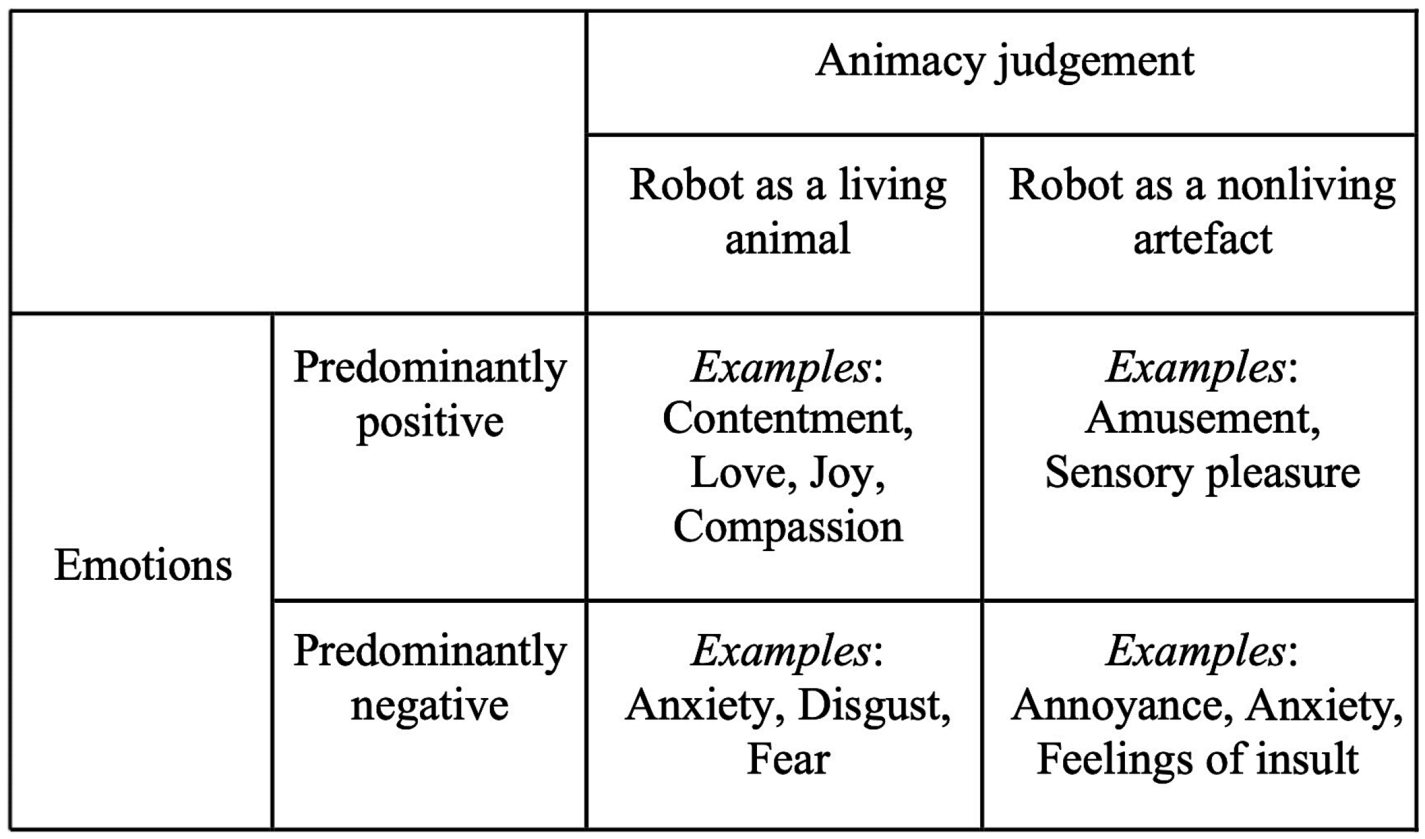
Analytical Model of Residents’ Emotions and Animacy Judgment of the Robotic Ambiguousness.

As described above, our findings indicate that this process of sensemaking is closely intertwined with an emotional orientation. The residents can—as we will show—respond with positive feelings (e.g., joy) if they, as Elisabeth, handle the robotic ambiguousness by making the animate judgment that the robot is a living organism. But residents might as well treat it as a fake animal, and still respond with positive emotions, such as amusement. On the other hand, they can express ambivalent or negative emotions, such as anxiety and annoyance, if they have trouble defining the ambiguous object or accepting the robot as a living animal. This is illustrated in Figure 1.

Research suggests that people often simultaneously experience positive and negative emotions, arguing for the need of a flexible approach to emotions (for instance Ekman, 1992; An et al., 2017). However, in this study we make a methodological choice to treat emotions analytically dichotomously—as positive or negative—in order to highlight typical responses to the robots found in our material. Our point of departure is that some of the emotions that characterize our everyday lives can be conceptualized as predominantly negative (such as anger, anxiety, or disgust), whereas others are of a more positive nature (such as happiness, contentment, or curiosity; Hviid Jacobsen, 2019, p. 3). Subjectively, people experience positive emotions as feelings that reflect a level of pleasurable engagement with the environment. Negative emotions, in contrast, reflect a general feeling of distress (Turner and Stets, 2006).

In the following sections, we will report on the residents’ different emotional stances toward the existential ambiguity of the robot animals.

### 5.2 Predominantly positive emotions

In the following, we will show how residents manage the built-in resistance of the robotic ambiguity by responding with positive emotions, that is, use these emotions as resources in their sense-making. First, we address situations in which the resident’s express emotions that indicate that they perceive the robot as a living animal. Thereafter we describe situations where the residents seem to judge the robot as a non-living artifact—yet responding with positive emotions when encountering it.

#### 5.2.1 Animate judgment: the robot as a living animal

Our first illustration of positive emotions connected to judging the robot as animate is an interaction moment between resident Viviane, a robot cat and one of the researchers (Marcus).

Viviane sits in a wheelchair and cuddles with the robot cat: “This cat must live here, they say. Because he is here almost all the time,” she tells Marcus. “Then you can stay here… you can sleep with me tonight,” she says to the robot cat. “You’re so warm. I know he’s in my bed sometimes, during the day, and it gets warm, and nice.” Directing her verbal communication both at the researcher by explaining the robot’s presence, and at the robot by offering her bed and complementing it (“You’re so warm”), Viviane involves both actors in the social situation but in different ways—providing information to the researcher and showing emotional closeness to the robot. She cuddles the robot and responds to its gestures both verbally and bodily. When the robot meows, she replies in a tender manner: “oh yes, little kitty,” and pats it. Viviane is comfortable and content in the company of the robot cat, which she conveys to the researcher:

I think he really seems kind this cat. You better stay inside. It’s not possible to be out tonight, no, ugh! (it is raining outside) We have two cats at home, but one is enough. They must have food several times a day. (…) This cat is very calm. He doesn’t move at all.

The robot cat meows, and Viviane responds with endearment: “Do you hear little kitty… oh you.” She pats the cat and looks at it. “Little kitty.” Again, her focus shifts between the researcher and the robot: “when the cats are kind, it’s so easy to have them. Just feed them and they’ll sleep,” she says to the researcher. And then she turns back to the robot: “And you are wearing a white shirt too.” (the cat is black with white spots on its chest, face, and paws) (Home 2).

Viviane’s first utterance “This cat must live here, they say” can thus be interpreted in relation to the fact that she has already defined the cat as real in previous interaction moments (“they say”). In this interaction moment, her judgment of the robot as a living animal is expressed by showing positive emotions. Her uncertainty (referring to mediated knowledge, “they say”) about the ambiguous robot is treated as unproblematic and verbal responses of contentment are made toward it: “You’re so warm. I know he’s in my bed sometimes, during the day, and it gets warm, and really nice” and “I think he seems really kind this cat.” Viviane also respond with compassion and a caring attitude toward the robot: “You better stay inside. It’s not possible to be out tonight, no, ugh” and talking in a loving voice: “Do you hear little kitty… yes you.”

The resident’s nonverbal expressions of positive emotions—cuddling the robot cat—trigger responses from the cat itself. When Viviane touches the pressure sensor while cuddling, the cat responds by meowing. An interaction chain has been set in motion where the human and the material object mutually respond to each other’s actions. Viviane seems to interpret the robot’s meowing as a positive reply to her cuddling, and by that her positive emotions toward the robot are enhanced through the ongoing interaction chain with the robot. This is evident in her continuing to articulate her positive emotions in the interaction with the robot cat.

Viviane’s assessment “This cat was very calm. He does not move at all” is interesting from a misinformation perspective. Her observation that the cat does not move may have led to her suspicion and, conversely, contributed to her perception of the cat as unreal. However, her other statements in this interaction can, on the contrary, be understood in terms of treating the cat as real, attributed with basic biological needs of food and sleep: “They must have food several times a day” and “When the cats are kind, it’s so easy to have them. Just feed them and they’ll sleep.” Thus, in response to a potential problem related to the robot’s immobility, compared to real cats, Viviane thus ends up interpreting this as a sign of calmness after creative problem-solving.

The second illustration of positive emotions associated with the judgment of the biomimetic robot as animate is an interaction moment where the actions and responses of the care worker plays a vital role in the resident’s interpretation of the robot cat as real. The observation involves Anna (resident), a robot cat and Molly (care worker, Home 2).

Molly has just entered the room with the robot in her arms and is presenting the robot cat to Anna, who responds in a cheerful manner: “Oh, little darling… oh dear!.” Molly carefully places the robot cat in Anna’s lap, making sure that she is receptive to it. While doing so, Molly continuously inserts positive replies, such as “oh yes,” to every utterance made by Anna. Like Viviane, Anna welcomes the robot cat with cuddles and warm greetings: “Oh, little cat,” “You can lie and sleep with me tonight.” Then she addresses Molly, “He’s so warm. But he lives here, right? It seems like he is always here.” Molly replies: “Yes, he lives here.”

In this situation, Anna speaks to the robot in loving and caring terms by calling it “little darling” and “little cat.” Molly confirms her positive emotions by repeatedly saying “Oh, yes.” When Anna directs her information-focused question to Molly (“He lives here, right?”), Molly, similar to how Flora interacted with Elisabeth, offers just enough information by simply confirming. Hereby, the care workers support the residents’ definition of the cat as real (and attributed a specific gender) and their positive emotions connected to this judgment. We understand the confirming responses of the care worker as a vital resource in the continuous interaction between the resident and the robot because they legitimize the residents’ emotional stance toward the robots. Thus, the social situation can be understood as a triadic relationship, involving three agents (resident, robot, and care worker) with different roles in the resident’s attempts to make sense of the ambiguous robot.

#### 5.2.2 Inanimate judgment: the robot as a nonliving artifact

Our first example of positive emotions and the judgment of the biomimetic robot as a nonliving artifact is an interaction moment between resident Harry, a robot cat, care worker Inga and a researcher (Marcus). In this social situation, the residents’ interpretive work of how the robot animal should be defined (real or not?) with the help of the dialog with the environment is very prominent.

The situation is as follows. Inga and Harry (Home 2) sit at a table in the common room, drinking coffee and talking. Inga invites Marcus to come and join the conversation, which he does. After a while the conversation is steered toward robot cats. Inga gets up to fetch the robot cat from another room and when she gets back, she lays it down on the table between Harry and Marcus, and says to Harry: “You can have it!.” Then she immediately moves away to help another resident. Harry and Marcus are left with the robot cat. Harry is hesitant and responds with caution. He looks at the robot with interest but does not touch it. Instead, he turns to Marcus and says: “No. I don’t want to touch it just in case. It looks so nice.” Then he says to the robot: “You are kind, right?” The robot is silent since no one is touching it. As an answer to his own question, Harry says: “Yes.” He turns to the researcher again: “It’s (the robot) a little different. It does not look like this (he turns his head), it’s just looking straight. They (the care workers) often say: you can have it. But no no no. I do not want it in case I lift it wrong or something. It’s looking. You see?” Harry shows Marcus by turning the cat a bit, so Marcus can look at its eyes. Harry continues: “Poor thing. It’s kind. I can put it back (he turns it back, so it is looking at him again). It’s so small and pretty.” Marcus asks: “Do you like animals?” and Harry replies: “Yes. but I’ve moved quite often so I haven’t had animals so… these are… what to say… fixed. They’re not mobile, but they’re still great.”

When Inga presents the robot cat to Harry, he responds with interest but also worry and suspicion, treating it as strange. By interacting with the robot as well as with the researcher he is trying to make sense of what the robot is in order to overcome the resistance its unfamiliarity offers: “You are kind, right?” He expresses positive emotions toward the robot by directing loving words toward it by calling it “nice,” “kind” and “great” as well as caring utterances like “poor thing” and “it’s so small and pretty.” His exploring, interpreting process of what the robot animal is, continues: “It’s a little different. It is not looking like this, it just looking straight.” Thus, Harry responds cautiously related to the cat’s potential vulnerability (“in case I lift it wrong or something”) as well as its unfamiliarity, not acting as real animals. Harry’s statements about the robot’s difference can be interpreted as having arrived at the definition that the cat is not real. However, his approach is still related to positive emotions: “…but they are still great.”

Our second illustration of positive emotions connected to perceiving the ambiguous robot as inanimate is a moment (Home 5) where Maja (care worker) introduces a robot dog to the residents Stig and Nora in the presence of a researcher (Clara).

The robot makes a ringing bark. Nora involves the robot by mimicking its pitch and nodding toward it. This practice of repeating sound is common as people interact with real pets (Harjunpää, 2022). Maja gazes at Stig, and Stig says: “what’s this?” with smiling voice followed by laughter, thus taking a stance of wonder rather than asking for information. This emotional orientation treats the ambiguous nature of the robot as positive rather than something to be solved. He asks: “Is it him doing that?,” which is followed by comments and laughter from the others, thus reciprocated as a comment rather than a question. After the laughter, Stig again comments on the dog’s barking, now treating it as a living being (“he”) and then addresses it: “Haha… You are a funny one hah hah hah.” Nora furthers this joyful situation by intercepting the interaction and saying: “I’m barking at you too…Woof!” She looks at Stig and laughs. Nora is talking to the dog, treating it as the recipient. However, she gazes toward the researcher, which indicates that her contribution is primarily for the benefit of the other participants. This generates a laughter from the researcher and Maja, and Stig smilingly offers a bark of his own.

In our interpretation Stig initially uses interaction as a resource to overcome the resistance he experiences in relation to the unfamiliar object, asking the other participants: “What’s this?” However, the unclear nature of the robot is treated as a source of joy rather than something that needs clarification and determination. While Stig’s approach to the robot does not show whether he thinks it is real or not, Nora later remarks to the researcher that “Stig thinks it is real.” In this sense, she responds to it as a living animal in this moment, while later showing awareness of its robotic nature.

Firmly establishing information about the robots’ character does, accordingly, not seem to be a requirement for (positive) emotional engagement in interactions involving robots. As one care worker (Home 5, Care worker 31) says: “We all have somewhat different inputs and for some it’s a real cat and for some it’s fake but it’s still fun.”

### 5.3 Predominantly negative emotions

In this section, we will show how the residents respond with negative emotions in their sense-making of the robots. First, we address situations in which the residents define and act upon the ambiguous robot as a living animal—yet expressing negative emotions when encountering it. Thereafter we acknowledge situations where the residents express negative emotions connected to the judgment that the robot is an inanimate artifact.

#### 5.3.1 Animate judgment: the robot as a living animal

Our first illustration of negative emotions related to perceiving the biomimetic robot as animate is an interaction moment where Betty (care worker) introduces a robot cat to Paul (resident). The situation takes place in the common room at one of the dementia homes (Home 2). One of the researchers (Marcus) is conducting an interview with two of the care workers (Betty and Susan). The conversation revolves around the use of technological devices and robots in dementia care. The participants are all sitting at a table with the robot cat on top. In the middle of the interview, Paul unexpectedly steps into the interview, curious of what we are doing. The care workers are quick to welcome Paul. They offer him a seat at the table next to them and involve him in the conversation.

Betty moves the robot cat closer to Paul and says: “Here, you can have a look at our cat!.” But Paul does not touch it. He just looks at it and replies: “that one can scare the crap out of anyone,” thus responding with fear. Both Betty and Susan respond to this comment as a joke, laughing. Paul does not, which demonstrates that he is not joking. In response, the care workers now treat Paul’s comment as related to fear by removing the robot cat from the table. When the robot is gone, Paul says: “It should be a dog instead.” Betty confirms his reply and answer: “Yes, we should have a dog.” And Paul repeats: “Better with a dog.” Susan is also quick to confirm his aversion toward the robot cat: “Yes exactly, cats can scare you. They may claw.”

Paul’s comment does not tell if he perceives the robot as real or not. However, we interpret Pauls’ declaring the problem is not connected to the robotic nature but rather to the “catness” (see Redmalm et al., 2022). Susan’s reply (“Yes exactly, cats can scare. They may claw”) also treats the robot as real by referring to his fear as justified given the potential danger of the general category “cats.”

The second illustration of negative emotions connected to the judgment of the robot as a living creature is from the same two care workers as above—Betty and Susan—but from a different occasion (Home 2). They are telling the interviewing researcher (Marcus) about an interaction moment with one of the residents and a robot cat, where the robot did not fill a helpful function. Betty informs the researcher about a resident that got worried when the robot meowed:

Yes, we had a lady whose cat… we couldn’t have the audio on because then she got worried… “My god, the cat needs help, and is hungry”… you know … a worry when it meows all the time. It needs help, what does it want, is it in pain? So, then we had it silent, then it worked great. She could feel it cooing.

Betty describes how the resident initially responded to the robot with negative emotions of anxiety and distress. In articulating these negative emotions, the resident simultaneously reveals her definition of the cat as real by granting it basic bodily needs, asking if it is hungry and in pain. When the staff as a response turned off the sound, the negative emotions, still connected to the perception of the robot as real, were replaced by positive emotions of contentment, evident in the resident being calmed by patting the (now silent) cat.

The researchers have been informed of similar experiences by other care workers. For instance, a care worker (Tina) at another dementia facility (Home 5) talks about a similar situation which involves a robot dog that barks. According to Tina, some residents find the robot dogs’ barking annoying. “So, we had to remove it because they went like this “shhhhhh” to the robot dog,” Tina says and puts her index finger to mouth to show the researcher that the resident tried to hush the dog. Tina knew that the residents she showed the robot to really like dogs, so she became a bit surprised at their reaction. “But it could be that dogs shouldn’t bark indoors,” she says. Tina’s interpretation of the hushing is that the residents reacted negatively, with annoyance, to the barking because the residents found the robot not well trained and its behavior in conflict with the social norm of a “good dog”.

Given that the residents’ attempts to reprimand the barking robot dog by hushing it only works with livings dogs, not getting this response may lead to feelings of being out of control. If they had viewed the robot as a machine, they had most likely understood that it would not learn anything from their attempts to train it. Instead, they might have asked the care worker to shut it off. However, they perceived the robot as a living dog, and acted upon it with displeasure and disapproval when barking.

#### 5.3.2 Inanimate judgment: the robot as a nonliving artifact

Finally, we show how negative emotions might be connected to judging the ambiguous robot as inanimate. The first example of this is recounted by a care worker (Home 4 and 5) who is also responsible for implementing technological devices in elderly care homes in her region. She regularly visits different elderly homes and to introduce robot cats and dogs, as well as other devices, to residents and staff, to see if they would be useful and of interest. It was in this capacity she was told of an interaction moment between a colleague of hers (Lucia), a resident (Dora), and a robot cat.

Lucia presented a robot cat to Dora informing her that it was a robot. Dora responded with interest and began exploring it. However, after patting the robot for a while, Dora noticed that the cat’s ears seemed oddly plastic. She suggested to Lucia that the cat should be taken to a veterinarian to have it checked. Lucia played along and agreed that it might be a good idea. However, upon continued and closer inspection, Dora realized that it was not a real cat. She then seemed disappointed and offended, and asked with an upset voice: “But why are you tricking me?”

The situation can be interpreted as the resident examining the robot cat in order to overcome resistance generated by the unfamiliar object by making sense of what it is. First, she declares her hypothesis that the cat is alive. This is indicated by the resident ascribing the robot cat bodily needs by suggesting that the ears are injured and in need of medical help. However, by continuing her defining process she ends up in the conclusion that the robot cat is not real after all. In connection to this definition the resident articulates negative emotions of disenchantment and annoyance, accusing the care worker of misleading her.

In our second example, the resident’s judgment of the biomimetic robot as a nonliving artifact is associated with negative emotions of being offended and ridiculed by the robot cat. In the following interaction moment (Home 2) June (care worker) sits at the dinner table beside Eva (resident), who has just finished eating breakfast. One of the researchers (Marcus) steps into the situation and sits down next beside them. Since Marcus was visiting the dementia home the day before, when June did not work, she asks Marcus if the robot cat was used yesterday. Marcus replies that it was used by different residents. June then says: “On Eva it does not work at all.” Eva hears her name and breaks in to wonder what we are talking about. June: “we are talking about you, Eva, and we are talking about that,” and she points to the robot cat, which is laying on a table next to us. Eva strongly denies that she uses the robot: “I do not know how many (others) that does it, but I have never used it before.” And she quickly adds that: “it is too childish.” Since there were several of the residents who were using the robot during yesterday’s visit, Marcus asks her: “I thought you too were playing with it yesterday?.” But again, Eva firmly objects: “no, I do not like it, it is too childish.” The resident clearly declares her judgment of the robot cat as inanimate and explains her unwillingness to use it by responding with negative emotions of being insulted by the offer of playing with a toy-like (“too childish”) cat. This way she demonstrates that she views the robot cat as a toy, and not as a living animal.

Our interviews with care workers about the residents’ responses to the robot animals contain more examples of the residents’ negative emotions of being offended by the robot animals since they do not perceive them as real animals. They acknowledge that some residents think that the robots are “ridiculous” (Home 3, Care worker 12), or “silly” (Home 1, Care worker 3). One care worker says that if a resident thinks a robot is a childish toy, it is crucial to respect that: “You know, it’s offensive, it’s as if you would get treated like a child, like ‘do you want a little doll in your lap?’” (Home 5, Care worker 34).

The interviews with care workers show that the negative emotions of being ridiculed by what they judge to be non-living animals often relate to unwillingness to use them, just as in the illustration above. One of the interviewees says: “The residents, they can, they have dementia too, but they think it’s ridiculous. ‘What do you think, I’m not a child, why do I have a stuffed animal here’, it’s like, ‘I want a real cat’” (house 5, Care worker 32). Thus, several of the interviewed care workers confirm the relationship between the residents’ emotions of being insulted by the biomimetic robot and their judgment that it is nonliving.

## 6 Concluding discussion

The study represents a novel endeavor to comprehend the significance of emotions as part of robotic misinformation within a care context. Having investigated how emotions become a resource for dealing with ambiguity related to animal robots in dementia care, we have shown that residents’ emotional expressions work as cues in collaborative sense-making with care workers. As residents display emotions, such as joy, happiness, contentment, irritation, and fear, care workers orient to the meaning of robot animals accordingly. This means that residents do not have to verbally define and request a specific treatment of a robot but that their emotional expressions instruct care workers to pragmatically allow for ambiguity as well as firm definitions of robot animals as real or fake. We also demonstrate that at times, care workers actively contribute to the establishment of misinformation about the nature of the robots when responding to the residents’ emotional expressions. For instance, they do so by referring to them as living creatures. A central finding is that emotions are not straightforwardly related to whether the robot is perceived as real or fake – whether residents fully understand the true nature of the robot, or if they are misinformed about the robot’s capacities. The lack of clear definitions does not hinder activities such as play, cuddling, or small talk involving robots. Instead, users often orient themselves to the robot as an ambiguous creature, prompting definitional work. These findings have two main implications.

Firstly, the analysis of residents’ orientations to the ambiguity of social robots highlights how persons with dementia take an active role in making sense of their world. Previous research has shown that confabulations—false statements about the world with no intention of lying—can be seen as one way in which persons with dementia produce order in face of a faltering memory (Örulv and Hydén, 2006). While such practices can produce difficult social situations because others may not agree with such worldviews, we see that robot animals allow for a less firm relation to truths and lies. Both care workers and residents ascribe functions to the robot animal in interaction with the environment, and pretense can be part of that interaction. Emotions become resources in this accomplishment, for example in how they allow care workers to show a general positive stance toward robots without defining them. And for residents, emotions can be understood as guiding a testing of hypotheses about the functions and identity of the robot.

Hypothesis testing, which is usually associated with rational thinking, can thus also be understood as an emotional practice: When experiencing resistance from the unfamiliar robot, the person with dementia tests different hypotheses about the robot animal’s function and meaning by expressing emotions (as significant gestures) in the interaction with others (e.g., care workers). Other people’s responses to such emotional orientations can in turn confirm or contradict the resident’s hypothesis. This is reminiscent of how legal professionals can draw on gut-feelings as they look for a true or defendable story (Minissale, 2023). While this group is highly trained to use their emotions in productive ways, both studies show that emotions provide resources for making sense in ambiguous situations, creating a legitimate reality together with others. Along other studies of interactions involving persons with dementia (e.g., Hydén and Antelius, 2017), this analysis thus contributes to a nuanced understanding of people with dementia and a critique of the commonly portrayed image of them as less than competent members of society and partners in interaction.

Secondly, our findings indicate that the principle of transparency has certain limitations when it comes to dealing with social robots’ ambiguity. It is noteworthy that, despite the varied settings where misinformation and technology pose pressing issues, the guidelines for the use of robot animals consistently advocate for transparency and information in a somewhat naive manner. This makes the guidelines difficult to apply for care workers in actual interactions with residents involving robot animals (e.g., Serrano-Puche, 2021). Rather than requiring clarity, transparency, and information, the use of robot’s places demands on care workers to navigate in a professional and responsive way to match dementia patients’ emotional orientations toward the robots. Thus, residents’ emotions do not only serve as resources in their sense-making process but also guide care workers in understanding how they should address the needs and sentiments of the residents, ensuring that the interaction with robots becomes a positive experience. While we have examined the responses of care workers to residents’ emotions, further research into the emotions of caregivers is warranted. This would contribute to a more comprehensive understanding of the emotional dynamics in the construction of robotic misinformation. Connected to a broader debate about deception in social robotics (see Blackman, 2013; Matthias, 2015; Leeson, 2017; Vandemeulebroucke et al., 2018), our findings align with the argument presented by Sharkey and Sharkey (2020) that attention must be given to “the deceived” (resident) and not only potential problems related to “the deceiver” (robot). Ethical guidelines in Sweden regarding robot use in care practice [Statens Medicin-Etiska Råd (SMER), 2014] state that patients should be informed about an introduced robot’s actual status and abilities. By contrast, our findings show that a flexible use, based on the ambiguity of robots, has the potential to create emotions of curiosity and joy (*cf.* Frezza et al., 2022). Our findings thus stress the need for further debates concerning guidelines for the use of robots in care work, as well as additional research on the significance of care workers’ emotions in the construction of robotic misinformation. A comprehensive framework for dealing with robotic misinformation in dementia care needs to be based on research that encompasses policy makers, developers, robots, care workers, as well as residents.

This study has stressed the importance of approaches to emotion and technology that examine robots’ functions in situated work. Using such an approach, this study points to a complex sociomaterial matrix involving triadic interactions between residents, robots, and care workers, in which emotions function as key resource to interpret, negotiate and establish a common understanding of reality.

## Data availability statement

The datasets presented in this article are not readily available because of ethical reasons. As residents in dementia care, the participants are in a vulnerable position. Requests to access the datasets should be directed to marcus.persson@liu.se.

## Ethics statement

The studies involving humans were approved by The Swedish Ethical Review Authority (Dnr 2020-04661). The studies were conducted in accordance with the local legislation and institutional requirements. Written informed consent for participation in this study was provided by the participants’ legal guardians/next of kin.

## Author contributions

MP: Conceptualization, Methodology, Project administration, Writing – original draft. ET: Conceptualization, Methodology, Writing – original draft. CI: Investigation, Writing – review & editing. DR: Investigation, Writing – review & editing.
